# Effect of pneumococcal conjugate vaccine availability on *Streptococcus pneumoniae* infections and genetic recombination in Zhejiang, China from 2009 to 2019

**DOI:** 10.1080/22221751.2022.2040921

**Published:** 2022-02-21

**Authors:** Xueqing Wu, Shanshan Zhao, Yan Jiang, Xi Xiang, Lihong Ge, Qiong Chen, Yanfei Wang, Jorge E. Vidal, Yunsong Yu

**Affiliations:** a Department of Infectious Diseases, Sir Run Run Shaw Hospital, Zhejiang University School of Medicine, Hangzhou, People’s Republic of China; b Key Laboratory of Microbial Technology and Bioinformatics of Zhejiang Province, Hangzhou, People’s Republic of China; c Regional Medical Center for National Institute of Respiratory Diseases, Sir Run Run Shaw Hospital, Zhejiang University School of Medicine, Hangzhou, People’s Republic of China; d Department of Clinical Laboratory, Shangyu People's Hospital, Shaoxing, People’s Republic of China; e Department of Clinical Laboratory, Affiliated Jinhua Hospital, Zhejiang University School of Medicine, Jinhua, People’s Republic of China; f Department of Clinical Laboratory, The Children’s Hospital, Zhejiang University School of Medicine, National Clinical Research Center for Child Health, Hangzhou, People’s Republic of China; g Department of Clinical laboratory, Affiliated Hangzhou First People's Hospital, Zhejiang University School of Medicine, Hangzhou, People’s Republic of China; h Department of Microbiology and Immunology, University of Mississippi Medical Center, Jackson, MS, USA

**Keywords:** *Streptococcus pneumoniae*, PCV availability, pneumonia, genetic variation, recombination

## Abstract

Pneumococcal pneumonia is one of the main reasons for child death worldwide. Pneumococcal conjugate vaccines (PCVs) are considered the most effective strategy for pneumococcal disease (PD) prevention, but how a pause in PCV vaccination affects the prevalence of PD or the genetic evolution of *Streptococcus pneumoniae* genetic evolution is unknown. Based on the unique PCV introduction timeline (vaccine unavailable during April 2015-April 2017) in China, we aimed to evaluate the effect of interrupted PCV availability on PD and pneumococcal genome variation. Pneumococcal isolates (n = 386) were collected retrospectively from eight sites in Zhejiang, China from 2009 to 2019 in which 184 pathogenic (isolates from sterile and infection sites) strains were identified. An interrupted time series analysis was conducted to estimate changes in PD and the recombination frequency of whole genome-sequenced strains was estimated via SNP calling. We found that both PD and pneumococcal genome variation were affected by interrupted PCV availability. The proportion (∼70%) of vaccine-type pneumococcal LRTI (VT-LRTI) in all LRTI cases decreased to ∼30% in the later PCV7 period and rebounded to ∼70% in children once PCV7 became unavailable in April 2015 (*p *= 0.0007). The major clone CC271 strains showed slowed (*p *= 0.0293) recombination frequency (decreased from 2.82 ± 1.16–0.72 ± 0.21) upon PCV removal. Our study illustrated for the first time that VT-LRTI fluctuated upon interrupted vaccine availability in China and causing a decreased of recombination frequency of vaccine types. Promoting a nationwide continuous vaccination programme and strengthening *S. pneumoniae* molecular epidemiology surveillance are essential for PD prevention.

## Introduction

*Streptococcus pneumoniae* (*S. pneumoniae*, pneumococcus) is one of the most common pathogens that cause infectious diseases such as otitis media, pneumonia, bacteremia, and meningitis, especially in children and elderly individuals, worldwide [1–3]. The World Health Organization (WHO) estimates that approximately 0.7-1 million children die every year from pneumococcal diseases (PDs), and most deaths occur in developing countries, including China [4,5]. Lower respiratory tract infections (LRTIs), including bronchitis, pneumonia, and bronchopneumonia, are the major PDs in Chinese children [6,7]. The WHO also reported that 81% of children who died due to PD presented with pneumonia [4]. It is critical for us to achieve close surveillance of pathogenic pneumococci and their related diseases, especially for LRTIs in Chinese children.

Vaccination is the best way to prevent pneumococcal infections [8]. The most recommended pneumococcal vaccine, pneumococcal conjugate vaccine (PCV) was introduced nationally or regionally in the past 20 years worldwide [9–12]. After vaccine intervention, national epidemiology studies in different countries have shown that the proportion of PDs caused by vaccine serotypes decreased significantly, while the proportion of nonvaccine serotype-related diseases increased in some countries, which supported the development of new vaccines [9,13–15]. The importance of the *S. pneumoniae* surveillance programme has been broadly emphasized but has only recently started in China.

The reason that pneumococcus- and/or PCV-related research is not considered important in China might be that neither PCV7 nor PCV13 is part of its national immunization programme, resulting in low vaccine coverage [16]. Studies in China reported no effect of PCV on pneumococcal serotype distribution [17]. However, none of those reports conducted investigations according to the unique situation of PCV availability in China. For instance, PCV7 became available (injection upon request and pay out of pocket) on the Chinese market in 2009 and was removed from the market in April 2015, whereas PCV13 has been approved for sale in China since May 2017. In March 2020, an in-country produced pneumococcal 13-valent conjugate vaccine (Woanxin) from Walvax Biotechnology Co., Ltd. was approved in China, which will be an alternative for Prevnar13 in the coming years [18]. Until then, Pfizer PCV (Prevnar and Prevnar13) was the only available pneumococcal conjugate vaccine for Chinese children. Therefore, a comprehensive analysis of pneumococcal disease, the epidemiology and evolution of *S. pneumoniae* strains during the PCV7-gap-PCV13 periods will guide future vaccination strategies.

In this article, we evaluated the change in PD and bacterial genetic variation induced by the PCV discontinuation in Zhejiang Province, China. Our findings refreshed the innate understanding that pneumococcal disease is barely affected by PCV in China and provided scientific evidence of vaccine-induced disease changes and bacterial genetic evolution that emphasizes the importance of continuous PCV vaccination.

## Methods

### Pneumococcal isolates and serotyping

*S. pneumoniae* isolates (representing all reported cases, n = 386) were collected from patients 0–96 years old during the PCV7 (January 2009 - March 2015, n = 266), PCV pause (April 2015 - April 2017, n = 57), and PCV13 periods (May 2017- December 2019, n = 63) in eight tertiary hospitals in Zhejiang Province, China. Serotyping was performed via latex and quellung reactions (for all isolates) using antisera purchased from the Statens Serum Institut (Copenhagen, Denmark) as well as whole-genome sequencing (WGS) analysis (for the isolates from patients under five years old, n = 128) using PneumoCaT (v1.2.1)[19] and SeroAB (v1.0.1).[20] Isolates with discrepant serotyping results (n = 3) among those three methods were resubmitted to two more quellung tests by different persons as the final determinant for their serotype (Supplementary Table 1).

### Clinical epidemiology

All clinical information of patients with positive pneumococcal cultures was retrospectively extracted from the medical records, including year, demographics, age, sex, underlying disease, and diagnoses (Table 1). The protocol of the current study was approved by the Sir Run Run Shaw Hospital Ethics Review Committee (Zhejiang University School of Medicine, 20201112-32). Pneumococcus-positive specimens included blood, cerebrospinal fluid, bronchoalveolar lavage fluid, sputum, and others. As shown in Table 2, isolates from blood, cerebrospinal fluid, bronchoalveolar lavage fluid, and infection sites (sputum/nasopharynx (NP)/oropharynx (OP) with respiratory infections, infection site secretion, etc.) were considered pathogenic pneumococci (n = 184). Patients diagnosed with pneumonia, bronchopneumonia, bronchitis, and lung infection were classified as LRTI positive. Those pneumococcal strains isolated from specimens of patients that had not been diagnosed with a pneumococcal disease were designated colonizing pneumococci (n = 191), for example, a strain isolated from sputum of a cancer patient that did not a clinically-confirmed respiratory infection was categorized as colonizing pneumococci.

**Table 1.** Characteristics of patients with S. pneumoniae culture positive.

Characteristics	Patients, N (%)
Overall	386 (100%)
Female	104 (26.9%)
PCV7 (2009-2015 March)	266 (68.9%)
PCV-Gap (2015 April - 2017 April)	57 (14.8%)
PCV13(2017 May - 2019)	63 (16.3%)
**Age (years, median ± SD)**	** **
0–5 (1 ± 1.4)	128 (33.2%)
6–64 (54 ± 16.2)	112 (29.0%)
≥65 (77 ± 7.4)	145 (37.6%)
N/A	1 (0.3%)
**Diagnoses**	** **
Meningitis	1 (0.3%)
Blood stream infection (BSI)	20 (5.2%)
Lower respiratory tract infection (LRTI)	155 (40.2%)
Upper respiratory infection (URTI)	7 (1.8%)
Otitis media	4 (1.0%)
Asthma	3 (0.8%)
Chronic obstructive pulmonary disease (COPD)	10 (2.6%)
Cancer	30 (7.7%)
Cardiac disease	13 (3.4%)
Non-meningitis cerebral disease (NMCD)	71 (18.4%)
Fever	11 (2.8%)
Others	59 (15.3%)
N/A	13 (3.4%)

PCV = pneumococcal conjugate vaccine; PCV7 = 7-valent PCV; PCV13 = 13-valent PCV; LRTI (Bronchitis, Bronchopneumonia, pneumonia, and lung infection); NMCD (Cerebral hemorrhage, Cerebral infarction, Cerebral trauma, and Cerebrovascular disease); N/A, information not available.

**Table 2.** Distribution of pathogenic and colonization pneumococcal isolates.

Age (years)	0–5	6–64	Over 65	Total
Pathogenic (n = 184)				
Invasive pneumococcal diseases (IPD)	12	6	4	22
Lower respiratory tract infection (LRTI)	100	14	40	154
Upper respiratory infection	4	3	0	7
Otitis media	2	1	0	3
Infection site	2	3	1	6
Total	114*	27*	43*	184*
Colonized (n = 191)				
Asthma	2	0	0	2
Chronic obstructive pulmonary disease	0	1	8	9
Cancer	0	8	22	30
Cardiac disease	0	5	8	13
Non-meningitis cerebral disease (NMCD)	0	41	29	70
Others	11	29	27	67
Total	13	84	94	191
N/A (n = 11)	1	2	8	11
Total				386

LRTI (Bronchitis, Bronchopneumonia, pneumonia, and lung infection); NMCD (Cerebral hemorrhage, Cerebral infarction, Cerebral trauma, and Cerebrovascular disease); *Cases were diagnosed with LRTI and blood culture positive, were counted both in IPD and LRTI (5, 1, and 2 cases in 0-5, 6-64, and over 65 groups, respectively); N/A, clinical data is not available.

### PCV coverage estimation and pathogenic pneumococcus rate in young children

It was difficult for us to obtain PCV coverage data in China due to a lack of PCV surveillance which is a consequence of PCV’s absence in our national immunization programme. However, PCV7 (Prevnar) (2009-2014) and PCV13 (Prevnar 13) (2017-2019) were the only available PCVs on the Chinese market before 2020. Therefore, the PCV coverage could be estimated by dividing the vaccine sale volume by three (three doses per child is considered an effective vaccination) and by the birth population of each year. We obtained PCV sale volume data for Zhejiang Province (2009-2019) from Pfizer Investment Co., Inc. The lot release data of PCV (2009-2019) were extracted from the open database of the National Institutes for Food and Drug Control of China [18]. The birth population data for Zhejiang Province (2009-2019) were obtained from the Zhejiang Provincial Bureau of Statistics (Supplementary Table 2) [21].

### Pathogenic pneumococcus isolation rate in young children

We calculated the vaccine-type pathogenic pneumococcal isolation rate and vaccine-type pneumococcal LRTI (VT-LRTI) rate in all LRTI cases in children under five years old from 2009 to 2019. Since the specimen collection was not evenly distributed in each year, we extracted a data point for the first half and the latter half of each year. For those years with less than three months of collection, less than ten pathogenic isolates were counted for one data point (2009, 2011, 2012, 2016-2019).

### Whole-genome sequencing analysis

Since young children were the most infected population in our study, genomic DNA of all pneumococcal isolates (single colony) from patients under five years old (n = 128) was prepared using a DNAmini kit (Qiagen, Valencia, CA, USA) and submitted to next-generation sequencing (NGS) using the Illumina HiSeq2000^TM^ platform. Illumina reads were mapped to a reference strain EF3030 (GenBank accession number NZ_CP035897) to make single nucleotide polymorphism (SNP) calls using the default parameters of Snippy (v4.4.5) [22]. Thereafter, the generated full alignment file was used to identify the recombination events via Gubbins (v2.4.1) [23] after five iterations, which utilized RAxML [24] to generate an initial maximum likelihood phylogeny followed by a scan statistic to identify recombination regions on every branch, and the phylogenetic tree was built in an additional step. The minimum number of base substitutions required to identify a recombination event was three. The ratio of SNPs caused by recombination and mutation (r/m) and recombination block number (re) were calculated yearly for each pathogenic strain. A paired-end fastq file for each isolate was assembled by Shovill [25], with a minimal length of 200 bp and a minimal coverage of 10x. The assemblies were then used as input for global pneumococcal sequencing clusters (GPSC) [26] assignment via PopPUNK [27] and *in silico* multilocus sequence typing (MLST) via PubMLST [28].

### Statistical analysis

An interrupted time series analysis (ITSA) model using a variance-centric approach was applied to estimate the effect of PCV on pathogenic pneumococci and VT-LRTI from 2009 to 2019. In China, it is difficult to determine the instant effect of PCV due to its absence in the routine immunization schedule. According to previous reports, PCV effectively reduced PD after five years of vaccination in the United States [29]. Therefore, we defined the fifth year (2013) of PCV7 licensure in China as the effective intervention start point, and April 2015 was the end point for our ITSA. ITS.analysis package v1.6.0 [30] was run in R v4.0.3 for such analysis using the defined variables (the proportion of pathogenic pneumococci or VT-LRTI). A type-2 sum squares analysis of covariance (ANCOVA) lagged dependent variable model was fitted to estimate the difference in means between interrupted and noninterrupted time periods, where *p *< 0.05 was considered statistically significant. We chose a time unit of three months and one year for those years with less than three months of collection or less than ten isolates to provide enough timepoints and enough cases for each timepoint. The correlation R value between PCV coverage and VT-LRTI rate was calculated using a two-tailed method. A strong correlation was defined when R^2^>0.6. To assess the difference in pneumococcal genetic recombination between time periods, we conducted a two-tailed Mann–Whitney test on each pair after data normalization. A two-tailed *p* <0.05 was considered statistically significant. Analysis was performed using GraphPad Prism v8.4.0.

## Results

### Clinical epidemiology

**Figure 1.**
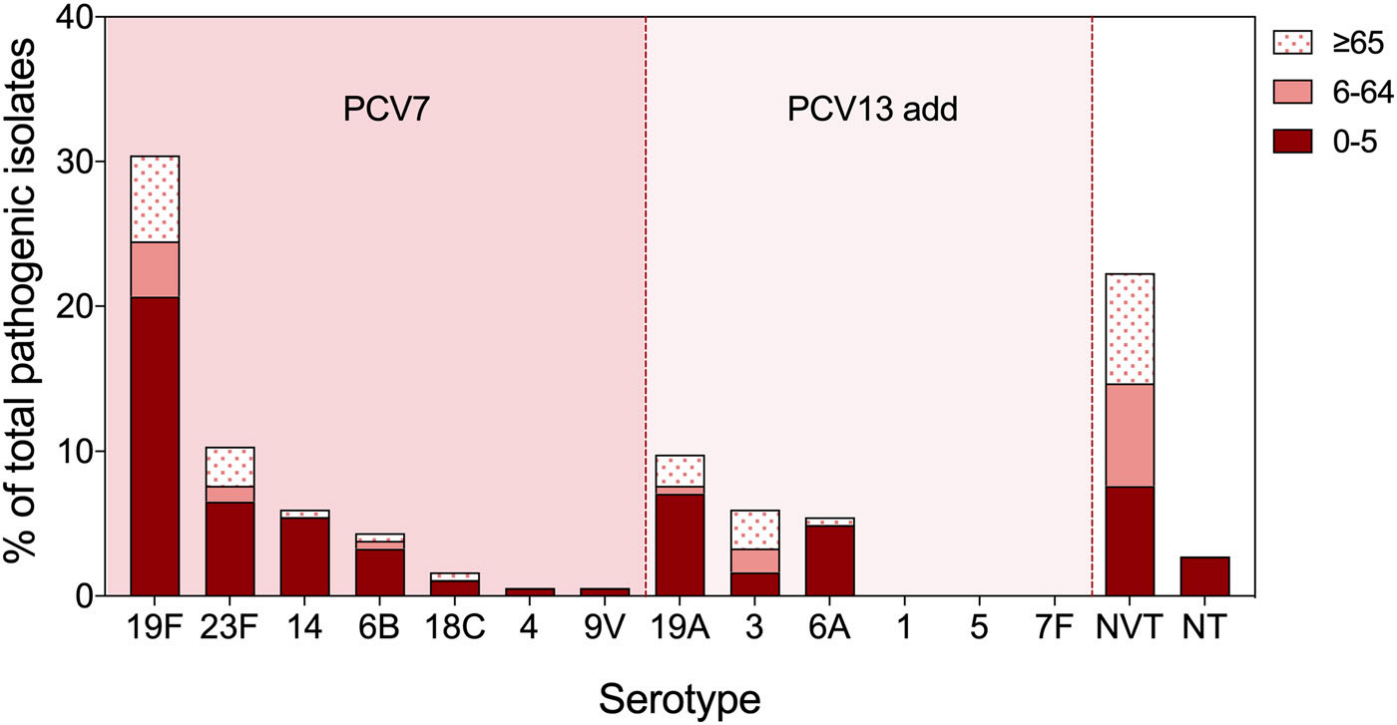
Serotype distribution of pathogenic pneumococcal isolates in different age groups. All pathogenic pneumococcal isolates were serotyped by latex and quellung reactions, where young children (<5 years old) related strains were also analyzed *in silico* by whole genome sequencing via SeroAB and PneumoCAT. The proportion of each PCV serotype, NVT (Non-Vaccine Type), and NT (Non Typeable) strains in all pathogenic pneumococcus were calculated in different age groups: 0–5 (red), 6–64 (pink), and, ≥65 (pink dots) years old.

Among all pneumococcal-positive individuals, 33.2% (128/386) were children under five years old, and 37.6% (145/386) were elderly individuals over 65 years old (Table 1). However, the majority of pathogenic pneumococcal isolates were collected from children under five years old (61.9%, 114/184), and LRTI isolates accounted for the most (87.7%, 100/114) (Table 2). Among all pathogenic isolates, the proportion from pneumococcal invasive disease was 11.8% (22/184), and more than half (54.5%, 12/22) were from children under five years old. Most of the colonizing pneumococci were isolated from individuals 6–65 years old (43.0%, 84/191) and over 65 years old (49.2%, 94/191) (Table 2). After serotyping, we found that PCV7/13-type strains accounted for most of the pathogenic pneumococci collected from children (84.2%, 96/114), among which 19F, 23F, 19A, 14, and 6A were the top five serotypes (Figure 1). Non vaccine type (NVT) pneumococcal strains accounted for 27.2% (105/386), of which 6D, 13, and 34 were the predominant serotypes (Supplementary Figure 2). Only 1.3% (5/386) of all isolates were nontypeable pneumococcal strains, which were all from children with LRTIs.

### VT-LRTI rebound in children due to a PCV pause

**Figure 2.**
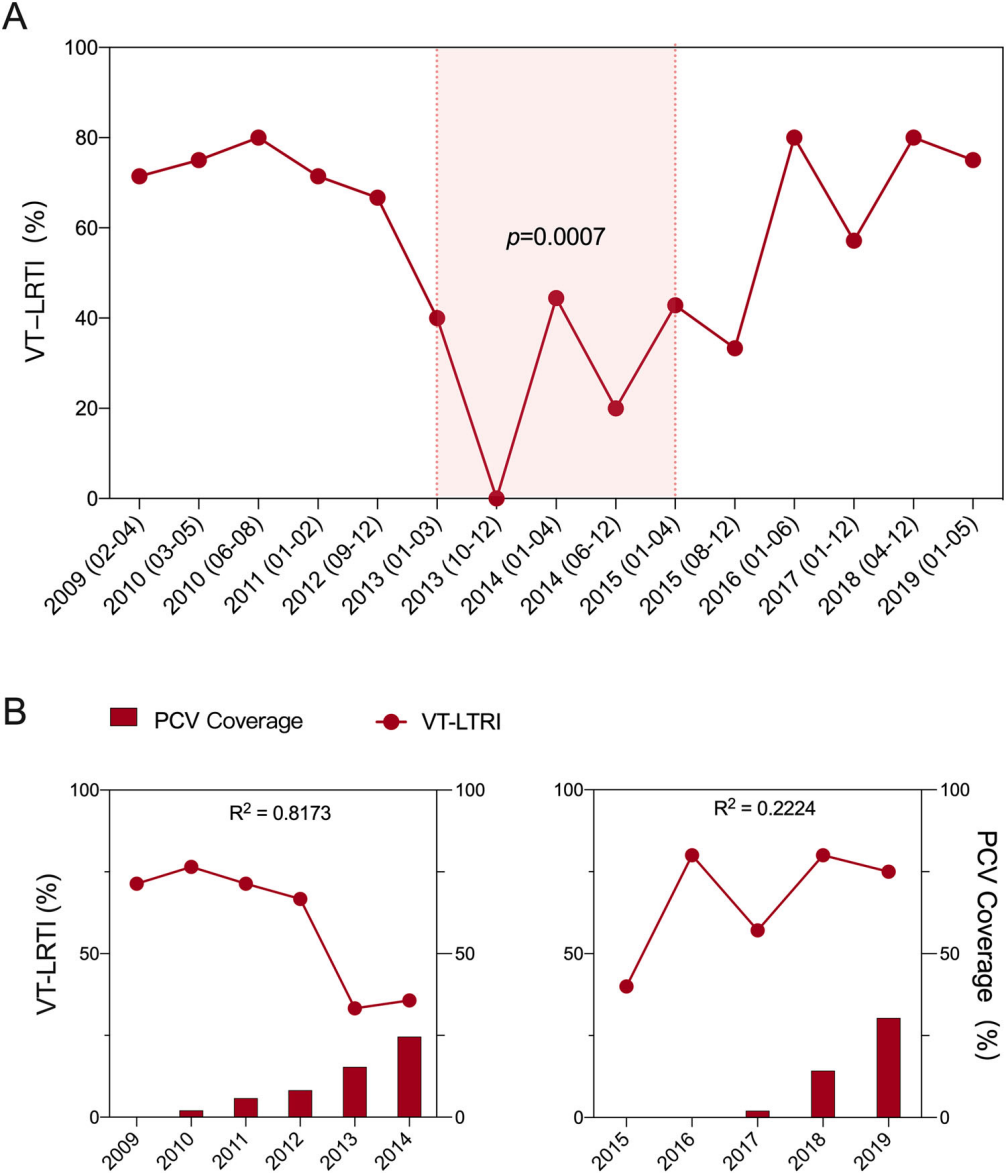
The ratio of vaccine type pneumococcus related lower respiratory tract infection (VT-LRTI) and its correlation with PCV coverage. A. The ratio VT-LRTI in children under 5 years old were calculated in all LRTI cases from 2009 to 2019. For the interrupted time serious analysis, two time-units in a year of 2010, 2013 2014, and 2015 were drawn for the first half and the latter half of each year. For those years have less than 3 months data or pathogenic isolates number less than 10 (2009, 2011, 2012, 2016, 2017, 2018, and 2019), only one time-unit was used for each year. The light red shaded time period (01/2013-04/2015) is the effective PCV7 intervention time. Statistically significate was determined when *p *< 0.05; B. The correlation of VT-LRTI rate (line) and PCV7 coverage (column) from 2009 to 2014; C. The correlation of VT-LRTI rate (line) and PCV7 coverage (column) from 2005 to 2019. A strong correlation was detected when R^2 ^> 0.6.

Since pathogenic pneumococci were mostly identified in children under five years old, we evaluated the effect of PCV on the pathogenic pneumococcal isolation rate as well as on the prevalence of LRTIs. Our results demonstrated that the overall pathogenic pneumococcal isolation rate did not change from 2009 to 2019 in Chinese children, nor was the proportion of total LRTIs (Supplementary Figure 1). However, the VT-LRTI rate was approximately 70% of all LRTI cases from 2009 to 2012 and decreased to approximately 30% at the fifth year after PCV7 approval in China, which remained similar until 2015. It increased again in 2016–80% of all LRTI cases and remained similar until 2019 (Figure 2 panel A). Overall, our interrupted time series analysis indicated that the VT-LRTI rate decreased when PCV7 was available and increased when it was unavailable (*p *= 0.0007) from 2009 to 2019 (Figure 2 panel A). As shown in Figure 2 panel B, the estimated PCV7 coverage in Zhejiang Province increased from 0% (2009) to 24.6% (2014) and dropped to 0% in 2015 and 2016. After the introduction of PCV13 in 2017, the estimated coverage of PCV13 increased to 30.4% in 2019. The correlation analysis suggested a strong negative correlation (R^2^=0.817) between the VT-LRTI rate and PCV7 coverage from 2009 to 2014. No correlation was observed between PCV13 coverage in 2017–2019 and the VT-LRTI rate (R^2 ^= 0.222).

### Molecular epidemiology of pathogenic pneumococci

**Figure 3.**
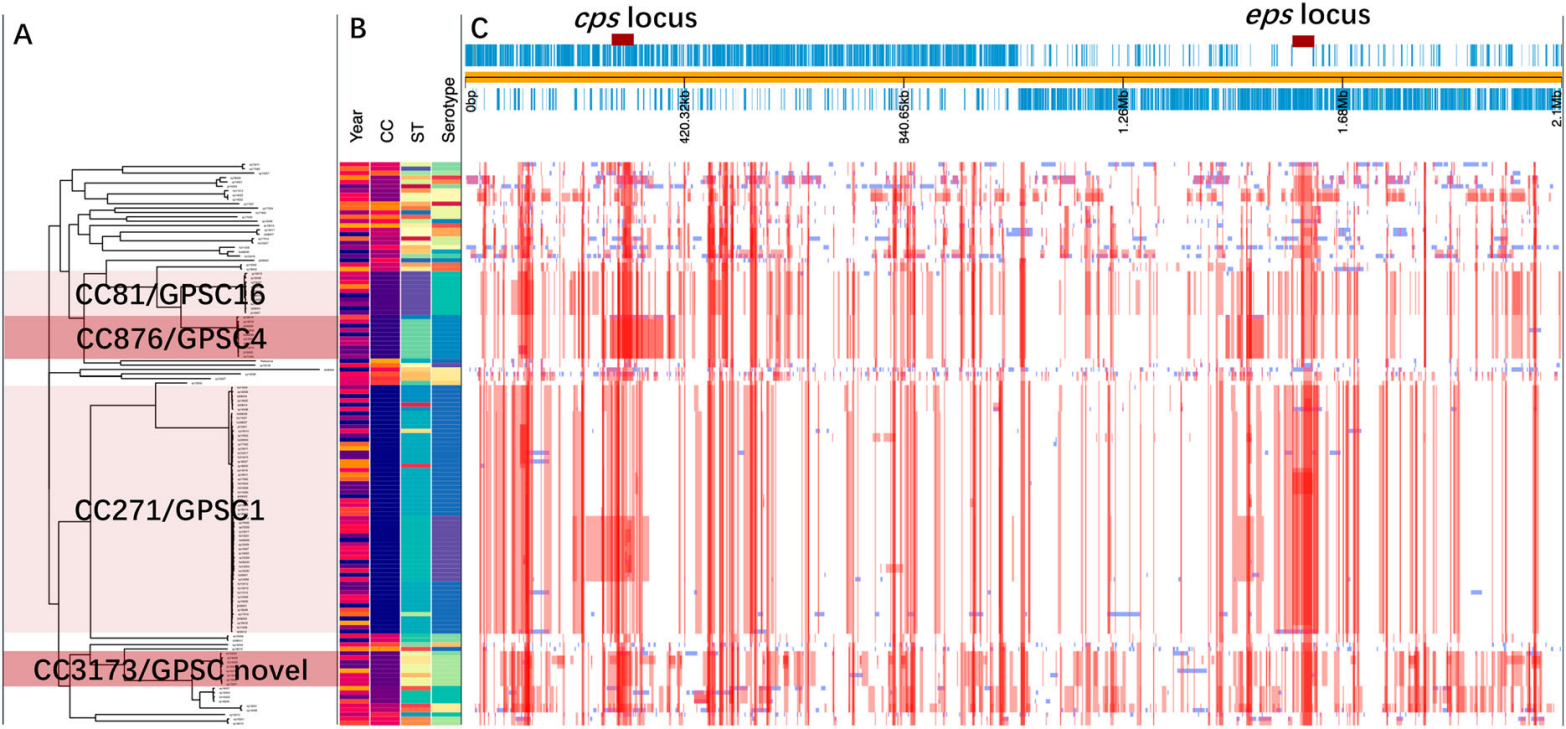
The phylogenetic tree and recombination events in all pneumococcal isolates from children under 5 years old. A. the phylogenetic tree of all pneumococcal isolates from children under 5 years old that was constructed in PopPUNK, where the four major clone complexes (CC) were shaded in light and dark pink; B. The metadata including isolation year, CC type, ST type, and serotype of all sequenced pneumococcal isolates; C. An annotated chromosome of the reference pneumococcal strain EF3030 with red marks of cps and eps locus (top of panel C). The main part of panel C shows the recombination events in all sequenced pneumococcal strains which were detected by Snippy and Gubbins. Red blocks represent the recombination blocks in each clone complex on an internal branch, which are therefore shared by multiple isolates, while blue blocks represent the recombination that occurred on terminal branches, which are unique to individual isolates. The whole data set was visualized in Phandango (https://jameshadfield.github.io/phandango/#/).

**Figure 4.**
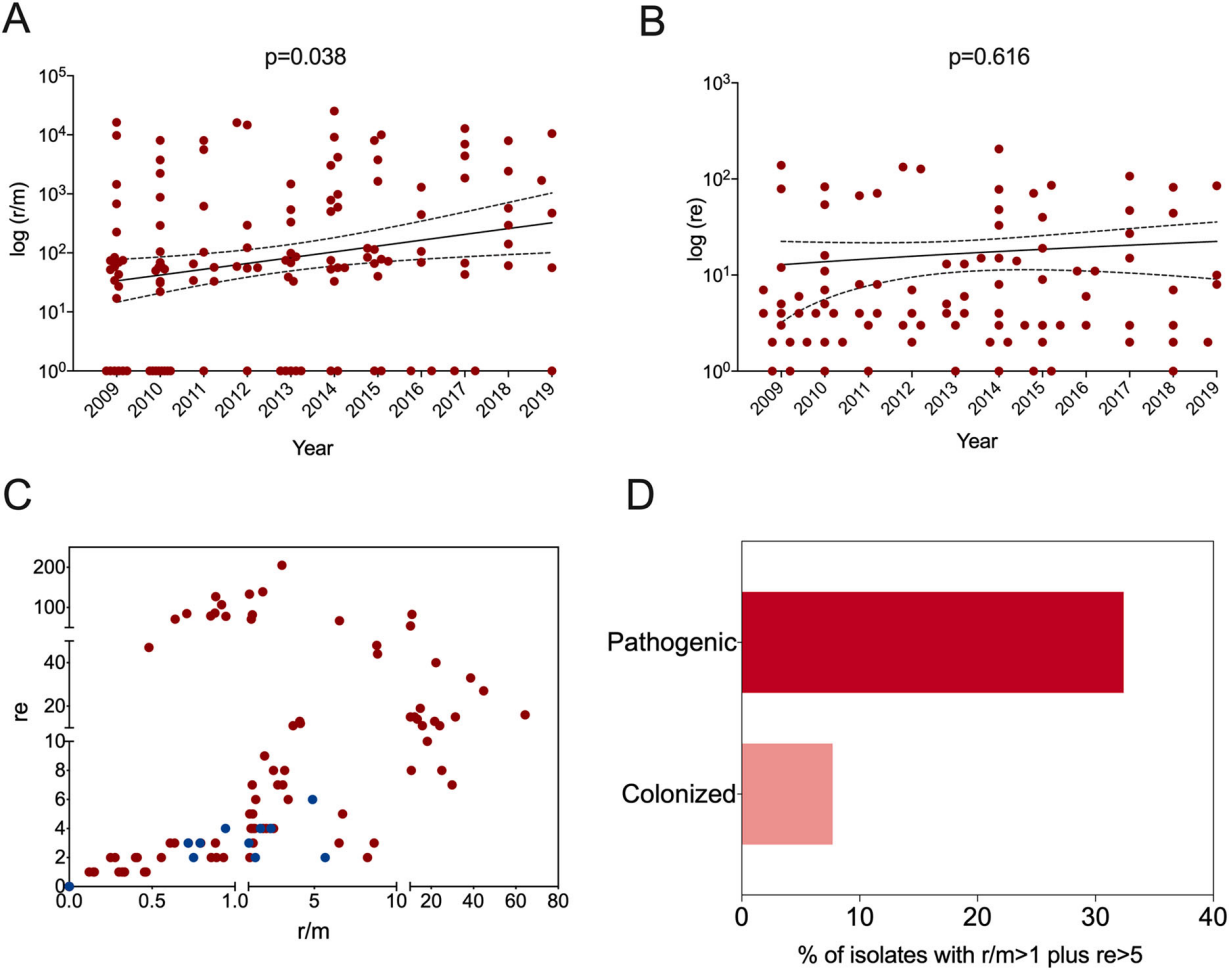
Recombination frequency (r/m) and numbers (re) of pneumococcal isolates collected from children under 5 years old. The ratio of SNP caused by recombination and mutation (r/m) (A) and recombination block numbers (re) (B) were determined in all pathogenic pneumococcal strains in children under 5 years old from 2009 to 2019. The data were presented as log (r/m) and log (re) for each isolate and a liner curve with 95%Cl was added for both. C. A two-dimensional Fig. of r/m and re of all pneumococcal isolates from children, where the blue dots represent colonized pneumococcal strains and red dots represent the pathogenic ones. D. The proportion of isolates with r/m > 1 and re > 5 in pathogenic and colonized pneumococcal strains.

To investigate the change in the pneumococcal genome from 2009 to 2019, we performed whole-genome analysis for all 128 strains from children under five years old (BioProject: PRJNA795524). Phylogenetic analysis identified four major clone complexes (CCs), 271 (GPSC1), 876 (GPSC4), 81 (GPSC16), and 3173 (a novel GPSC) (Figure 3), among which CC271 was the most prevalent clonal complex and included serotype 19F and 19A strains, accounting for 46.1% (59/128) of all child isolates. A serotype switch event can be determined when more than one serotype appears in a single sequence type (ST), which was observed for only ST3173 that contained serotypes 6A and 6B. SNP analysis indicated that recombination events were common among all clones, especially around the capsule polysaccharide (*cps*) and exopolysaccharide (*eps*) loci, which encode pneumococcal polysaccharide capsule biosynthesis factors (Figure 3). A significant increase in r/m was observed from 2009 to 2019 (Figure 4 panel A), which was not affected by vaccine availability. No re change was detected (Figure 4 panel B). A two-dimensional graph of r/m and re for all isolates showed that colonized strains (blue points in Figure 4 panel C) tended to have undergone less recombination. It is believed that the genetic variation of each strain was introduced by recombination rather than spontaneous mutation when r/m>1. Among all pneumococcal strains from children under five years old, 32.4% (37/114) of pathogenic strains presented a high recombination character (r/m>1 and re>5), while only 7.7% (1/13) of colonizing isolates showed similar tendencies (Figure 4 panel D).

### Fluctuating recombination activities caused by PCV discontinuation

**Figure 5.**
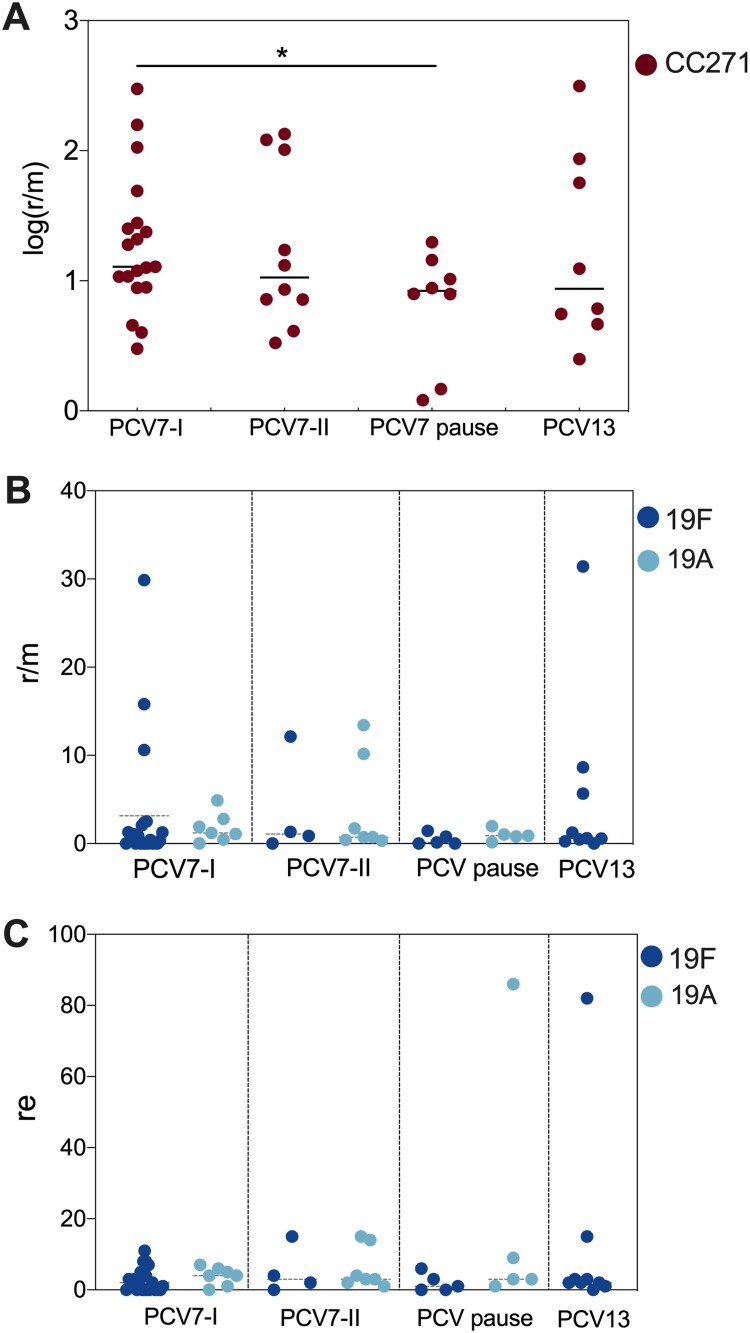
Recombination frequency (r/m) and numbers (re) of CC271, 19F, and 19A in different PCV time period. A. The normalized (log) r/m value of CC271 in PCV7-I (2009-2011), PCV7-II (2012-2014), PCV-pause (2015-2016), and PCV13 (2017-2019), “*” indicate a significant difference between PCV7-I, and PCV pause groups (*p *= 0.0293); B and C. The r/m and re value of serotype 19F (dark blue dots) and 19A (light blue dots) strain in the above four time periods.

Increased recombination activity in children was observed from 2009 to 2019, and no PCV interruption-induced change was detected in general. However, it is different for the most prevalent clonal complex CC271, which contains serotype 19F (PCV7 type) and serotype 19A (PCV13 added type) strains. As shown in Figure 5 panel A, CC271 strains exhibited a significantly decreased recombination frequency in the vaccination pause period compared with that in the PCV7-I period (2009-2011). After a detailed analysis of serotype 19F and 19A strains (Figure 5 panel B), we found higher r/m values in serotype 19F strains (up to 31.42) than in serotype 19A strains (up to 12.13) in the time periods with PCV on market. A high re pneumococcal strain in the serotype 19A group was detected in the PCV pause period and in the serotype 19F group after PCV13 was licensed (Figure 5 panel C). In the PCV13 period (2017-2019), we had data for only serotype 19F strains, as no 19A strains were isolated during this time.

## Discussion

China has dramatically improved child survival, with a tremendous decrease in pneumococcal deaths from over 28,000 to approximately 7,000 per year, [29] but pneumococcal pneumonia is still the most identified reason for PD-related deaths in Chinese children under five years old (72%) [4,29]. Although it is the best intervention to prevent PD, PCV was reported to have no clear influence on pneumococcal serotype distribution or related infections in Chinese children [17,31]. This phenomenon might be due to the low coverage of PCV in China, which is a consequence of PCV’s absence in our national immunization programme [32]. However, in this study, for the first time, we reported a rebounded VT-LRTI rate in children due to a PCV discontinuation from 2015 to 2017 in Zhejiang Province, China. Moreover, we also found that the predominant clone, CC271, showed slowed recombination activities during the PCV pause period.

Among all pneumococcal isolates in this study, the majority of pathogenic strains were isolated from children under five years old, among which LRTI isolates accounted for the largest proportion. These results are in accordance with commonly accepted findings that *S. pneumoniae* is prevalent in young children and causes infectious diseases, especially LRTIs [33]. Similar to previous reports [17,34] on serotype distribution in China, we also found that PCV7/13 serotype strains are the dominant pathogenic pneumococci in young children, among which serotypes 19F, 23F, 19A, 14 and 6A were the top five isolated serotypes, demonstrating a foreseeable positive effect of PCV13 on PD in the future.

Since the most pathogenic isolates were collected from young children with LRTIs, it is worth closely examining the effect of PCV discontinuation on pneumococcal LRTIs in children. In our study, the ratio of pathogenic pneumococcal isolates did not change throughout these years, nor did that of LRTI strains, which suggested no general influence of PCV on PD in China [32]. However, the proportion of VT-LRTIs was clearly decreased after five years of PCV7 application in China, which is similar to the outcome of a significant decrease in PD among children after routine use of PCV7 in the United States since 2000 [29]. After a pause in PCV7 availability from 2015 to 2017, the VT-LRTIs rebounded, which has not been reported before. Certainly, the interrupted availability of PCV-induced pneumococcal respiratory diseases rebounds in children.

PCV coverage is never clearly reported in China due to the absence of this vaccine in its national immunization programme, resulting in a diversity of PCV uptake depending on the educational and economic level of different regions of the country. For example, 0.0% PCV7 coverage in Yiwu in 2014 [35] and 10.1% coverage in Shanghai in 2015 were reported [31]. No previous studies have shown the PCV coverage trend from 2009 to 2019. As a well-developed province, in our estimation, Zhejiang Province showed a rapid increase in PCV coverage that reached 24.6% in 2014 and 30.4% in 2019. The increase in PCV coverage was negatively correlated with the VT-LRTI rate in the PCV7 period. As reported previously, PCV7 administration decreased the pooled prevalence of pneumococcal nasopharyngeal carriage from 25% to 14% in China [34]. The decrease in pneumococcal carriage following PCV7 administration might be a contributor to the observed negative correlation in our study. We also found that this correlation did not persist after the removal of PCV7 and approval of PCV13 from 2017-2019. Although the coverage of PCV13 was higher in 2019 than that of PCV7 in 2014, the positive influence of vaccines may need reinitiation due to the PCV discontinuation. Certainly, the goal of pneumococcal vaccination is to reduce the burden of PD caused by any serotype. The lack of a significant decrease in the isolation rate of pathogenic pneumococci post-PCV13 introduction might also be due to the time to market being too short. Given that the majority of disease-related pneumococci in young children are PCV13-type strains and that there was a decrease in VT disease during the PCV7 period, it is promising that the effect of PCV13 will improve in coming years if we apply a continuous vaccination strategy.

Pneumococci acquire DNA from the environment and other strains by transformation and homologous recombination, which ensures that they evade environmental stress, such as that due to PCV vaccination [36]. Serotype switching or vaccine escape are representative examples of this strategy after PCV intervention, which has been widely reported in different countries [37]. To the best of our knowledge, the evolution of pneumococcal strains via genetic recombination due to PCV pressure has not been studied in strains isolated in mainland China. From an overall perspective of our genetic analysis, serotype switching was only observed in strains of the clonal complex ST3713. This may be due to the low vaccination coverage and/or the PCV discontinuation; but certainly, it indicates that serotype switching is not a common occurrence yet in mainland China. In contrast, in Hong Kong, a geographically adjacent region, serotype switching events occurred in several STs, for example, serotype 19F/23F in ST81, serotype 19F/19A in ST320, and serotype 6B/6C in ST76 [38], which were due to routine vaccination with PCV7 since September 2009 [39].

Regarding vaccine intervention, an increased r/m since the approval of PCV in China showed that the evolution of *S. pneumoniae* was already initiated by PCV even with relatively low vaccine coverage. However, the major clone CC271 behaved according to PCV availability; for instance, the strains showed decreased recombination frequency when PCV coverage dropped, and serotype 19F strains were found to be more active than serotype 19A strains. These findings are consistent with previous studies demonstrating that pneumococci rapidly incorporate genes in their chromosomes to evade environmental stresses, in this case, vaccine intervention [36,40]. Our findings illustrated a quick evolutionary response of pneumococci to interventions that will lead to vaccine escape or serotype switching in a short time.

We found that pathogenic pneumococci exhibited more chromosome recombination than colonized pneumococci in young children. It has been reported that pneumococcal recombination is associated with capsule size, carriage duration, and carriage prevalence [36]. First, the majority of defined pathogenic pneumococci in our study were collected from sputum specimens and therefore selection had already happened, and hence increased their recombination frequency; selection can be a result of long-term colonization in the patient’s respiratory tract. Additionally, to be a virulent strain, Pneumococcus also needs to adapt to different environments to induce infections, which requires an evolutionary advantage via the most convenient method of genetic modification, recombination.

Our study has also a number of limitations. First, high heterogeneity was noticed in the number of isolates from each site. Since the current study was not a prospective pneumococcal surveillance programme, this variation might be explained by differences between study sites in bacterial strain laboratory storage. However, laboratory personnel at all sites have a similar educational and economic levels, and we believe that this variation does not affect the main conclusion of our study. Second, the invasive PD incidence might be underestimated in our study, primarily because a blood culture is not a routine test for potential pneumococcal cases in China. Therefore, we avoided drawing any biased conclusions on invasive PD cases. Third, none of the study sites applied the recommended clinical pneumococcal isolation procedure (enrichment broth culture) [1] in any Chinese hospital. Direct blood agar culture of specimens is the main method utilized in our clinical laboratories. This method of isolation may dramatically decrease the number of pneumococcal isolates and miss pneumococcal infection cases. Hence, we cannot exclude the bias of PCV influence on disease burden due to an underestimation of PD cases. We believe that drawing a conclusion based on the relative disease ratio is more reliable.

## Conclusion

In this retrospective observational study, we collected pneumococcal isolates from eight sites in Zhejiang, China. We found that a PCV pause eliminated the advantage of previous PCV vaccination for PD. Pneumococcal genetic variation via recombination was also changed due to PCV availability in China. Vaccination with pneumococcal vaccines will significantly contribute to PD prevention in China if we encourage continuous immunization with PCV13 and/or a new generation of pneumococcal vaccines and conduct comprehensive surveillance programmes on PD and molecular epidemiology.

## Contribution of authors

XW wrote the manuscript. XW, SSZ, and YJ contributed equally to the data management and analysis. YSY, JEV, and XW designed the study and data analysis. JEV also contributed to the manuscript writing. SSZ and QC contributed to the collection of specimens and data management. SSZ, XX, LHG, and YFW were responsible for lab work including serotyping and whole genome sequencing of all isolates.

## Supplementary Material

Supplemental Material
