# Supplementary material for: Effect of pneumococcal conjugate vaccine availability on Streptococcus pneumoniae infections and genetic recombination in Zhejiang, China from 2009 to 2019
Source: Emerg Microbes Infect. 2022 Feb 21;11(1):606–15. doi: 10.1080/22221751.2022.2040921 (PMC8865111; doi:10.1080/22221751.2022.2040921)
Supplement: Supplemental Material [file TEMI_A_2040921_SM1376.zip › Suppl files/Supplementary Figure legends.docx]

**Supplementary Figure legend**

**Supplementary figure 1. Pathogenic pneumococcus and lower respiratory infection rate (LRTI) in children under 5 years old in last decade.**

The pathogenic pneumococcus isolation (A) and LRTI rate (B) in young children under 5 years old. For the interrupted time serious analysis, two time-units in a year of 2010, 2013 2014, and 2015 were drawn where each of them contains 3 months data. Only one time-unit was drawn in the years have less than 3 months data (2009, 2011, 2012, 2016, 2017, 2018, and 2019). The light red shaded time period (2013-2014) is the effective PCV7 intervention time. Statistically significate was determined when *p*<0·05.

**Supplementary figure 2. The serotype distribution of non-vaccine type *S. pneumoniae* in Zhejiang province, China from 2009 to 2019.**

Non vaccine type pneumococcal strains were serotyped by Latex reaction and quellung test to identify the specific serotype for each strain. The Y-axis represent the number of isolates and X-axis show the serotype of each strain. The most prevalent serotypes (n=8) were marked as red.
