# Supplementary material for: Effect of pneumococcal conjugate vaccine availability on Streptococcus pneumoniae infections and genetic recombination in Zhejiang, China from 2009 to 2019
Source: Emerg Microbes Infect. 2022 Feb 21;11(1):606–15. doi: 10.1080/22221751.2022.2040921 (PMC8865111; doi:10.1080/22221751.2022.2040921)
Supplement: Supplemental Material [file TEMI_A_2040921_SM1376.zip › Suppl files/Supplementary Tables.docx]

| **ID** | **Quellung 1^st^** | **PneumoCAT** | **SeroAB** | **Quellung 2^nd^/3^rd^** |
| --- | --- | --- | --- | --- |
| sy13052 | 6B | 6A-D mix | 6A | 6B/6B |
| hz10030 | 32F | 33F/A | 32F | 32F/32F |
| jx09002 | 29 | 19F/29 | 29 | 29/29 |

**Supplementary Table 1. Isolates with discrepancy serotyping results between Quellung test, WGS analysis via PneumoCAT, and SeroAB.**

**Supplementary Table 2. National lot release of PCV, sale volume, and birth population data in Zhejiang.**

|  | **PCV7** | | | | | | **PCV-gap** | | **PCV13** | | |
| --- | --- | --- | --- | --- | --- | --- | --- | --- | --- | --- | --- |
| Year | 2009 | 2010 | 2011 | 2012 | 2013 | 2014 | 2015 | 2016 | 2017 | 2018 | 2019 |
| Sale volume (injections) | N/A | 34,349 | 90,889 | 136,584 | 253,323 | 427,316 | N/A | N/A | 42,211 | 269,725 | 555,549 |
| Lot release (injections) | 309,576 | 585,738 | 385,300 | 808,672 | 660,553 | 1,198,530 | N/A | N/A | 715,415 | 3,847,534 | 4,754,514 |
| Birth population (10k) | 52.63 | 55.08 | 51.66 | 55.36 | 54.93 | 57.80 | 58.10 | 62.40 | 67.00 | 62.80 | 60.90 |
