# Supplementary figures and images for: Effect of pneumococcal conjugate vaccine availability on Streptococcus pneumoniae infections and genetic recombination in Zhejiang, China from 2009 to 2019
Source: Emerg Microbes Infect. 2022 Feb 21;11(1):606–15. doi: 10.1080/22221751.2022.2040921 (PMC8865111; doi:10.1080/22221751.2022.2040921)

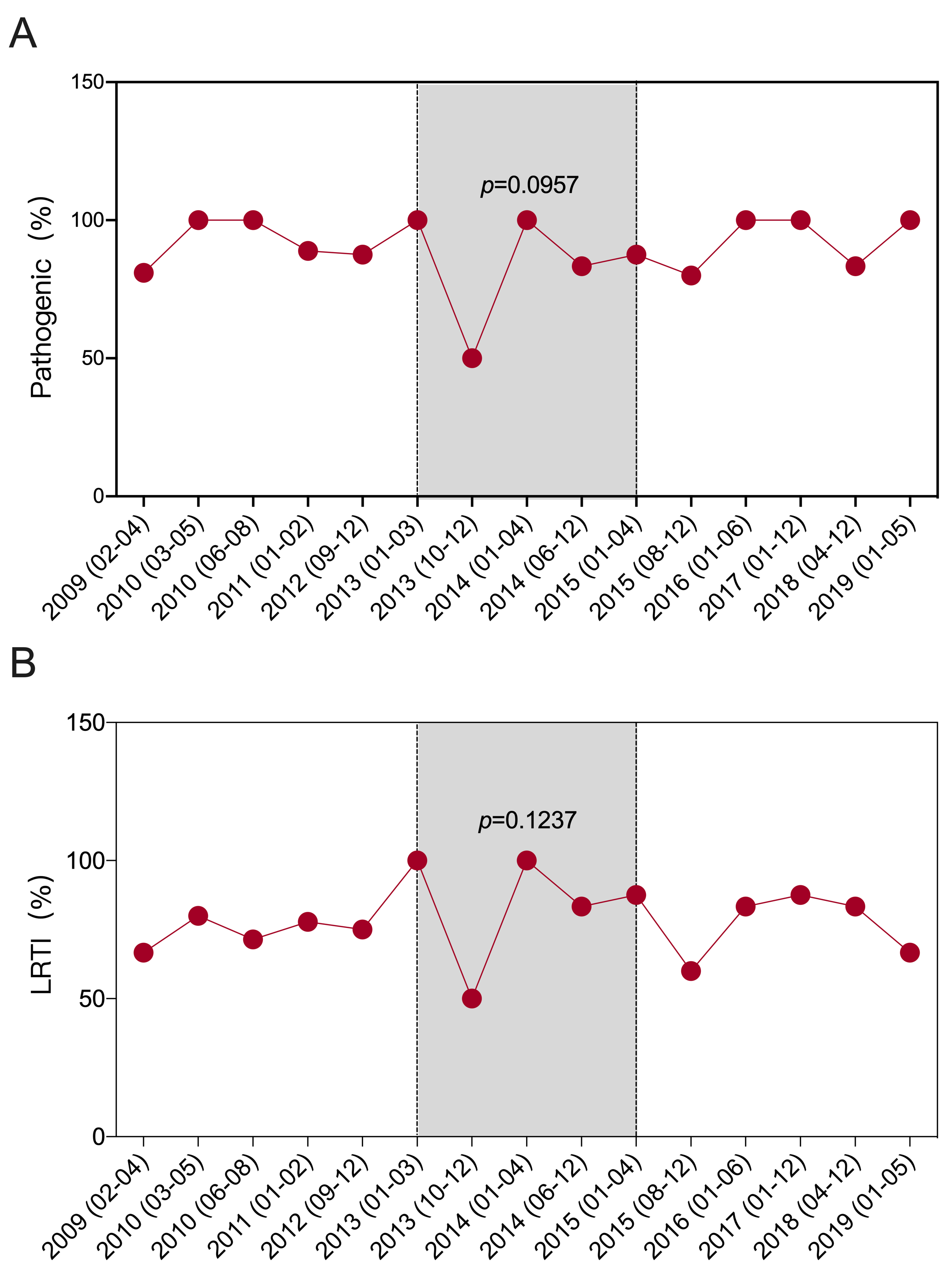

Supplement: Supplemental Material [file TEMI_A_2040921_SM1376.zip › Suppl files/Sup_fig1.tiff]

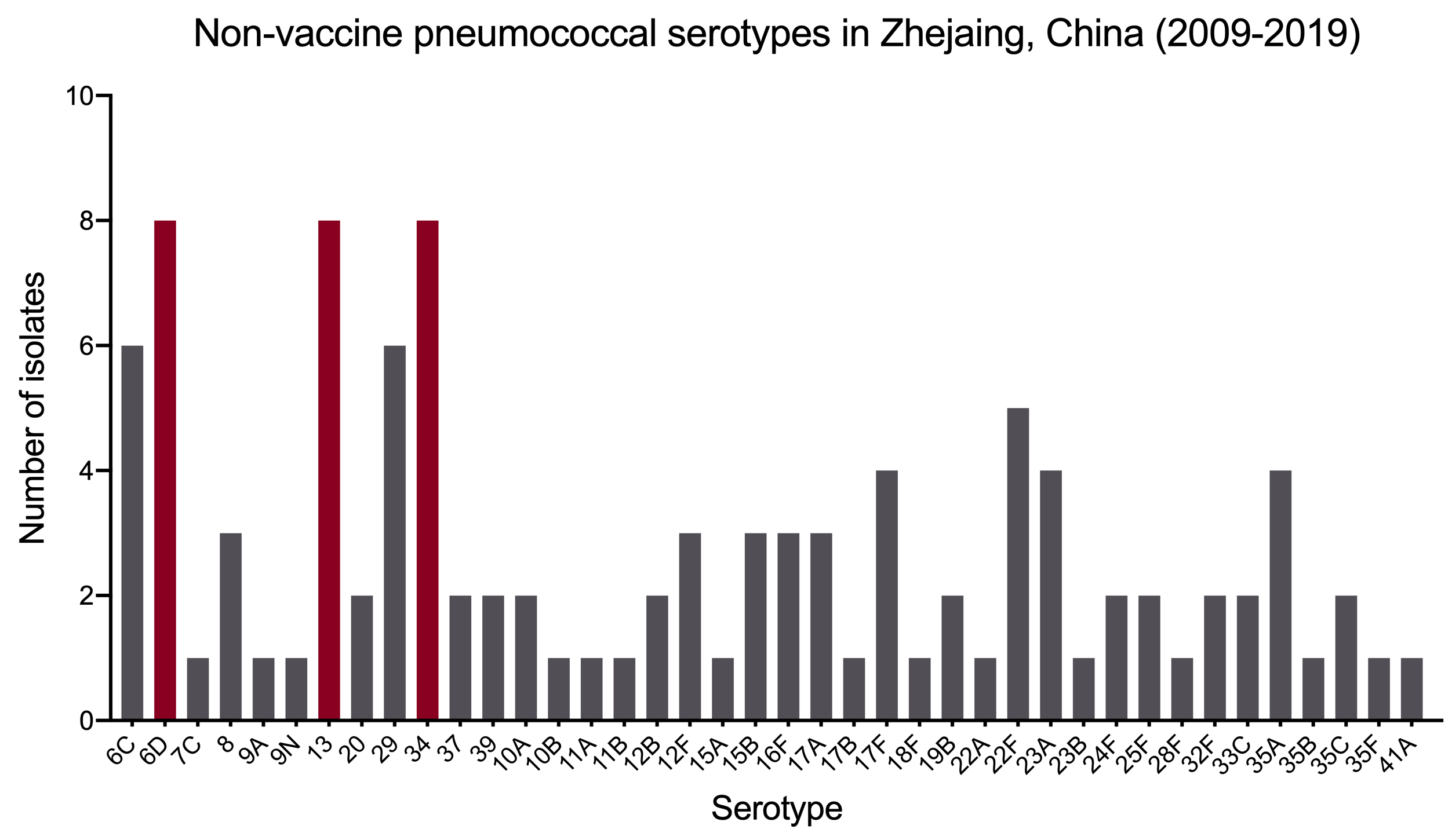

Supplement: Supplemental Material [file TEMI_A_2040921_SM1376.zip › Suppl files/Sup_fig2.tiff]
